# Venous thromboembolism prophylaxis for hospitalized medical patients, current status and strategies to improve

**DOI:** 10.4103/1817-1737.69104

**Published:** 2010

**Authors:** Hikmat Abdel-Razeq

**Affiliations:** *Department of Internal Medicine, Division of Hematology and Medical Oncology, King Hussein Cancer Center, Amman, Jordan*

**Keywords:** Deep vein thrombosis, heparin, low molecular weight heparin, pulmonary embolism, thromboprophylaxis

## Abstract

Venous thromboembolism (VTE), comprising life-threatening pulmonary embolism (PE) and its precursor deep-vein thrombosis (DVT), is commonly encountered problem. Although most patients survive DVT, they often develop serious and costly long-term complications. Both unfractionated heparin and low molecular weight heparins significantly reduce the incidence of VTE and its associated complications. Despite the evidence demonstrating significant benefit of VTE prophylaxis in acutely ill medical patients, several registries have shown significant underutilization. This underutilization indicates the need for educational and audit programs in order to increase the number of medical patients receiving appropriate prophylaxis. Many health advocacy groups and policy makers are paying more attention to VTE prophylaxis; the National Quality Forum and the Joint Commission recently endorsed strict VTE risk assessment evaluation for each patient upon admission and regularly thereafter. In the article, all major studies addressing this issue in medical patients have been reviewed from the PubMed. The current status of VTE prophylaxis in hospitalized medical patients is addressed and some improvement strategies are discussed.

Venous thromboembolism (VTE), comprising life-threatening pulmonary embolism (PE) and its precursor deep-vein thrombosis (DVT), is a commonly encountered problem. The annual incidence of VTE in western countries is 2–3 per 1000 inhabitants.[1,2] This incidence has remained relatively unchanged since early 1980s.[3] In the absence of prophylaxis, some studies have reported VTE in 10–26% of patients admitted to a general medical ward.[4] Although most patients survive DVT, they often have serious and costly long-term complications; almost one-third of patients with DVT suffer from venous stasis syndrome (postphlebitic syndrome), manifested by painful swelling and recurrent ulcers.[5] In view of the clinically silent nature of VTE, the incidence, prevalence and mortality rates are probably underestimated. Given the above, VTE imposes major health and financial burden for the whole community.

Though VTE is a common problem, fortunately, it is preventable. Identifying high-risk patients and the application of suitable prophylactic measures are the best ways to prevent VTE and its associated complications, the most serious one being PE which may happen suddenly leaving not much time for the physicians to act.[6] Almost one-third of the patients with PE die within 1 week and one-fourth die suddenly.[7]

## Risk Factors

Medically ill patients are a very heterogeneous group; not all of them need thromboprophylaxis. Several surveys and descriptive studies have tried to identify special groups of patients who are at a higher risk for VTE. One special feature of medical patients is their age; most of the medically ill patients are elderly. Risk of thrombosis increases sharply with age; from approximately 1 in 10,000 people per year for those younger than 40 years to 1 in 100 people per year for those 75 years and older.[8,9] Thus, as the average population age increases in any community, the prevalence of VTE increases too. Among medical patients; myocardial infarction, ischemic stroke and cancer are associated with a high risk for VTE; these clinical entities are usually studied separately since they require unique prophylactic measures. In a multiple logistic regression analysis, Alikhan *et al*,[10] showed that age older than 75 years, cancer, previous VTE, acute infectious disease and chronic respiratory disease were all independent risk factors for VTE. Previous VTE had the highest odds ratio [OR: 2.06; 95% confidence interval (CI): 1.10–3.69]. Other significant risk factors for VTE in medical patients include confinement to a hospital or nursing home, extremity paresis, central venous catheterization and heart failure.[11] In females, additional risk factors include hormonal therapy with oral contraceptive pills,[12] hormone replacement therapy, and selective estrogen receptor modulators like Tamoxifen and Raloxifene.[13] Diseases like myeloproliferative disorders, especially essential thrombocythemia, nephrotic syndrome, paroxysmal nocturnal hemoglbinuria, Bahçet syndrome and inflammatory bowel disease, are some of other medical illnesses associated with higher risk of VTE.[11]

## VTE Prophylaxis: Does it Work?

In the absence of prophylaxis and using different radiological screening tests, DVT rates have been reported in a significant percentage of medical patients.[14–16] Using autopsy studies, 2.5% of 200 medically ill patients followed up prospectively with no prophylaxis, were observed to have fatal PE.[17] Comparing unfractionated heparin (UFH) with placebo in medically ill patients, the rate of radiologically detected DVT was reduced significantly without bleeding complications.[15,18]

LMWH was also compared with placebo in medically ill patients; in a randomized double-blind trial, Dahan and colleagues[14] used enoxaparin once daily or placebo in 275 medical patients over the age of 60 years. The incidence of radiologically proven DVT was reduced from 9% in the placebo group to 3% in the group of patients receiving enoxaparin (*P* = 0.03). Except for injection site hematoma that was more frequent in the LMWH group, there was no difference in major bleeding in the two groups.

The MEDENOX trial[16] (prophylaxis in MEDical patients with ENOXaparin) was a randomized, double-blind, placebo-controlled study designed to study the value of LMWH at a two-dose level, in reducing the incidence of VTE in medical patients. Patients were older than 40 years with expected hospital stay of at least 6 days and had been recently immobilized for 3 days or less. Patients were admitted with acute heart failure, New York Heart Association (NYHA) class III or IV, or acute respiratory failure (but not requiring immediate ventilatory support). Other patients included in this study had one of the following three medical conditions: acute infectious disease without septic shock, acute rheumatic disorder or an active episode of inflammatory bowel disease and at least one predefined VTE risk factor (age > 75 years, cancer, previous history of VTE, obesity, varicose veins, hormone replacement therapy and chronic heart or respiratory failure). A total of 1102 patients were enrolled, evaluable patients (*n* = 866) were divided into three treatment groups: placebo, enoxaparin at a dosage of 20 mg and 40 mg, all given once daily subcutaneously. The incidence of radiologically proven VTE was reduced from 14.9% in the placebo group to 5.5% in the group that received enoxaparin at 40 mg (*P* < 0.001), a risk reduction of 63%. However, there was no significant difference in the incidence of VTE between the group that received low dose enoxaparin (20 mg) and the placebo group. Additionally, there was no significant difference in major bleeding among the three groups.

In another study, Prospective Evaluation of Dalteparin Efficacy for Prevention of VTE in Immobilized Patients Trial (PREVENT), Leizorovicz and colleagues[19] randomized 3706 medical patients to receive either 5000 IU of dalteparin once daily or placebo for 14 days. The patients underwent a bilateral compression ultrasound on day 21 to search for asymptomatic proximal thrombi. The incidence of VTE was reduced from 4.96% in the placebo group to 2.77% in the dalteparin group, an absolute risk reduction of 2.19% or a relative risk reduction of 45% (relative risk: 0.55; 95% CI: 0.38–0.80; *P* = 0.0015). The observed benefit was maintained at 90 days. The overall incidence of major bleeding was low but higher in the dalteparin group (nine patients; 0.49%) compared with the placebo group (three patients; 0.16%).

A meta-analysis of seven randomized trials comparing prophylactic UFH or LMWH versus placebo in medically ill patients, excluding acute myocardial infarction or ischemic stroke, was performed.[20] The primary end points were DVT detected at the end of the treatment period and clinical PE; other end points include death and major bleeding. More than 15,000 patients were included. A significant decrease in DVT and clinical PE was observed with heparins as compared to control (risk reductions: 56 and 58% respectively, *P* < 0.001 in both cases), without a significant difference in the incidence of major bleedings or deaths. Above discussed studies indicate that VTE prophylaxis with UF or LMWH is safe and effective in high-risk medical patients.

## Underutilization

Despite the evidence demonstrating significant benefit of VTE prophylaxis in acutely ill medical patients, several registries have shown significant underutilization. In the DVT-Free registry; 5451 patients with ultrasound-confirmed DVT, including 2892 women and 2559 men, from 183 United States (US) centers were enrolled in prospective registry. The most frequent co-morbidities were surgery within 3 months, immobility within 30 days, cancer and obesity. Of the 2726 patients who had their DVT diagnosed while in the hospital, only 1147 (42%) received prophylaxis within 30 days before diagnosis.[21]

In a national multicenter Canadian survey study (CURVE study), the medical records of patients in 20 teaching and 8 community hospitals were reviewed to assess the adherence to the established 6^th^American College of Chest Physicians (ACCP) consensus guidelines for VTE prophylaxis. Of the 4124 medical admissions screened over the study period, 1894 patients (46%) were eligible for study inclusion. The median age of this cohort was 70 years. Forty-one percent of patients were bedridden for more than 24 hours and 31% had one or more identified risk factors for VTE. Overall, some form of thromboprophylaxis was administered only to 23% of all patients and to 37% of patients who were bedridden for more than 24 hours. However, appropriate prophylaxis was given to only 16% of the patients.[22] Similar findings were also reported in the IMPROVE study in which 15,156 high-risk medically ill patients with a median age of 71 years and a median length of hospital stay of 8 days enrolled at 52 hospitals; less than 60% of the patients received appropriate VTE prophylaxis.[23]

## Choice of Prophylactic Agent

UFH, LMWH and mechanical devices like elastic stocking or Intermittent Pneumatic Compression (IPC) are the available options. More recently, new agents like pentasaccharides have also been introduced for this indication too. Though there are no enough good data to discuss the use of different mechanical modalities in hospitalized medical patients, several studies, including a Cochrane review, showed that the use of graduated compression stocking reduced VTE in hospitalized patients after surgery by about 50%.[24,25]

Using a large, multi-hospital, US database, McGarry et al. identified high-risk medical patients aged ≥40 years hospitalized for ≥6 days for an acute medical condition; 479 patients received enoxaparin prophylaxis, while the majority (2837 patients) received UFH. The incidence of VTE was 1.7% in the enoxaparin group versus 6.3% in the UFH group (RR = 0.26; *P* < 0.001), a relative risk reduction of 74% in favor of enoxaparin. The risk of major bleeding and mortality was similar in both the groups.[26]

Several other studies have looked into the issue of the ideal thromboprophylactic agent, but a few were controlled and randomized. In one prospective study, the Thromboembolism Prophylaxis in Internal Medicine with Enoxaparin (PRIME) study; Lechler and colleagues[27] randomized 959 immobilized medical patients to receive enoxaparin 40 mg once daily or UFH 5000 units three times daily, both given subcutaneously. The primary end points were objectively confirmed PE or DVT. VTE was diagnosed in one of the 393 (0.3%) patients on LMWH and in 5 of the 377 (1.3%) patients receiving UFH (*P* = 0.2); major bleeding tended to be less with LMWH (0.4%) versus 1.5% with UFH.

The efficacy and safety of the LMWH enoxaparin was also compared to UFH in the prevention of venous thromboembolic disease in patients with heart failure or severe respiratory disease. In a multicenter, controlled, randomized study (The PRINCE), 665 patients received either enoxaparin (40 mg once daily) or UFH (5000 IU 3 times daily) for 10 ± 2 days. The primary end point was a thromboembolic event up to 1 day after the treatment period. The incidence of thromboembolic events was 8.4% with enoxaparin and 10.4% with UFH. Authors concluded that enoxaparin was at least as effective as UFH, with a one-sided equivalence region of –4% (90% CI: –2.5 to 6.5; *P* = 0.15). Enoxaparin was associated with fewer deaths, less bleeding and significantly fewer adverse events (45.8% versus 53.8%, *P* = 0.044).[28]

More recently, the use of LMWH was compared to that of UFH in stroke patients, a group of medically ill patients with high risk for VTE. The PREVAIL study was an open-label trial comparing the efficacy and safety of enoxaparin versus UFH in preventing VTE events in patients with acute ischemic stroke confirmed by CT who had sufficient paralysis that they were unable to walk unassisted. A total of 1762 patients were enrolled within 48 hours of stroke symptom onset and randomized to receive either 40 mg of enoxaparin given daily subcutaneously or 5000 IU of UFH given twice daily for 10 ± 4 days, depending on when the patient was able to ambulate. The patients were followed for a period of 90 days. The primary efficacy end point was a composite of symptomatic or asymptomatic DVT and symptomatic or fatal PE during the treatment period. This trial showed a 43% reduction in the primary end point (RR: 0.57; 95% CI: 0.44–0.76; *P* = .0001) with enoxaparin versus UFH.[29] In the previously discussed meta-analysis,[20] nine other trials comparing UFH with LMWH for prophylaxis in 4669 hospitalized patients showed no significant differences between the two treatment groups in the rates of DVT, clinical PE or mortality. However, LMWH reduced by 52% the risk of major hemorrhage (*P* = 0.049). Given the above studies, one can conclude that LMWH is at least as effective as UFH when given three times a day. However, because of the convenience of once a day injection and the possibility of lower incidence of bleeding, we favor LMWH for the highest risk medical patients while UFH twice a day, or LMWH, can be offered for lower risk patients.

## Duration of Prophylaxis

Many medically ill patients continue to have risk factors for VTE even after hospital discharge. Emphasis on better bed utilization and early hospital discharge have moved more high-risk medical patients out of hospitals to ambulatory-based treatment where VTE prophylaxis is not routinely practiced. The benefits of extended out-of-hospital thromboprophylaxis have already been demonstrated in patients undergoing major orthopedic and elective cancer surgery. Extended out-of-hospital thromboprophylaxis is recommended by many international guidelines for this surgical population.[30]

The concept of extended out-of-hospital prophylaxis was recently tested in acutely ill medical patients with prolonged immobilization. The Extended Clinical prophylaxis in Acutely Ill Medical patients study (EXCLAIM) compared the efficacy and safety of extended prophylaxis (28 ± 4 days) versus the standard enoxaparin regimen (10 ± 4 days) in acutely ill medical patients with recent reduced mobility.[31] Patients aged ≥40 years with recent reduced mobility for up to 3 days with either level 1 mobility (total bed rest or sedentary) or level 2 mobility (level 1 mobility with bathroom privileges), and who had at least one of the pre-defined medical conditions were enrolled. Pre-defined medical conditions included heart failure; NYHA class III or IV, acute respiratory insufficiency without need for mechanical ventilation; and other acute medical conditions including, but not limited to ischemic stroke, infection without septic shock, rheumatic disorders, inflammatory bowel disease or active cancer. The study failed to show significant advantage of extended prophylaxis in the study group, so the inclusion criteria were amended to include higher risk group. Patients with level 2 mobility had to have at least one other risk factor for VTE (age > 75 years, a history of VTE or diagnosed cancer). Compared to placebo, extended-duration enoxaparin reduced VTE incidence (2.5% vs. 4%; absolute risk difference favoring enoxaparin, -1.53%[95.8% CI, -2.54% to -0.52%]). However, enoxaparin increased major bleeding events (0.8% vs. 0.3%; absolute risk difference favoring placebo, 0.51%[95% CI, 0.12% to 0.89%]). The benefits of extended-duration enoxaparin seemed to be restricted to women, patients older than 75 years, and those with level 1 immobility. This study demonstrates that extended out-of-hospital prophylaxis might be considered for higher risk medical patients; however, lower incidence of VTE should be weighed against the higher risk of bleeding before making such decisions.[32]

## Pentasaccharides, the New Class of Anticoagulants

Fondaparinux (AriXtra, GlaxoSmithKline) is the first agent of a new class of selective factor Xa inhibitors (pentasaccharides). This new synthetic compound, with no animal-sourced components, has been designed to bind selectively to a single target in the plasma, Antithrombin (AT), which inactivates factor Xa, thus resulting in a strong inhibition of thrombin generation and clot formation.[33] This drug was first approved for venous thromboembolic prophylaxis in patients undergoing major orthopedic procedures. More recently, fondaparinux was also approved for the active therapy of PE and DVT.[34,35]

Fondaparinux was also tried as a prophylactic agent in medically ill patients. In one study, the ARTEMIS trial, 849 patients aged 60 or more, hospitalized for acute cardiac, respiratory, infectious or inflammatory diseases, and considered to be at moderate risk of VTE were enrolled. These patients were randomized to receive either fondaparinux at 2.5 mg dosage subcutaneously once daily or placebo starting within 48 hours of admission and continued for 6–14 days. A bilateral venogram was performed on days 6–15. Patients randomly assigned to fondaparinux had a significant reduction in the incidence of VTE as compared to the placebo group (5.6% versus 10.6%: relative risk reduction, 47%; *P* = 0.03). Five patients in the placebo group had fatal PE, compared to none in the fondaparinux group. Major bleeding occurred in one patient in each group.[36] This study illustrates again the real need for VTE prophylaxis in this group of patients. Additionally, it showed that fondaparinux is effective and safe for this indication too. However, one can argue that a lower incidence of VTE could have been achieved at a much lower cost utilizing agents like UFH or LMWH. This study would have been of more value if it had compared fondaparinux with these agents.

## Can We Do Better?

Despite its proven efficacy, VTE prophylaxis is clearly underutilized. Many reasons can explain this consistent underutilization. Lack of physician awareness and agreements on published VTE prophylaxis guidelines and the underestimation of the risks in this group of patients continue to be important barriers. However, lack of a validated VTE risk assessment model able to group medical patients into different risk categories is probably the most import barrier. While decisions on how and when to start VTE prophylaxis are easier to make in surgical patients, such decisions are more difficult in a very heterogeneous group of medical patients; majority are elderly with many diverse and complex medical problems. This was evident by findings of the ENDORSE study in which 58.5% of the 11,613 surgical patients at risk were given prophylaxis compared to only 39.5% of the 6119 at risk medical patients.[37]

Recently, many health advocacy groups and policy makers are paying more attention to VTE prophylaxis. The National Quality Forum (NQF) recently endorsed strict VTE risk assessment evaluation for each patient upon admission and regularly thereafter.[38] Additionally, the Joint Commission is in the process of applying a new standard that will hold medical centers accountable for ensuring that VTE prophylaxis is addressed. This standard mandates that a VTE prophylaxis measure is in place within 24 hours of hospital admission, otherwise, a risk assessment and contraindications for prophylaxis should be documented for each and every hospitalized patient.[39] Recently, Maynard and Stein.[40] have published their experience and recommendations following their extensive efforts to better utilize VTE prophylaxis in high-risk patients. Such recommendations are worth careful attention and are summarized in Table 1.

**Table 1:** Strategies to improve VTE prophylaxis, in medically ill patients

Support by hospital administration for better VTE prophylaxis initiative
Establishment of “VTE Prophylaxis Multidisciplinary Team”; this team should standardize the process of providing VTE prophylaxis facilitates implementation of guidelines, audit and monitor the results, report regularly to hospital administration or a “Quality Council”
Better guidelines:
Simple, yet efficient in daily use; two to three levels of VTE risk are enough
Provide clear link between risk level and prophylaxis choice
Provide guidance to manage patients with contraindications
Continuous education and training of all health care providers

In a new innovative method to enhance the rate of prophylaxis, researchers from Brigham and Women’s hospital developed a computer program linked to the patient database to identify consecutive hospitalized patients at risk for VTE. The program randomly assigned 1255 eligible patients to an intervention group, in which the responsible physician was alerted to a patient’s risk of VTE and 1251 patients to a control group in which no alert was issued. The physician was required to acknowledge the alert and could then withhold or order prophylaxis. The primary end point was clinically diagnosed, objectively confirmed DVT or pulmonary embolism at 90 days. Prophylactic measures used in this study included graduated compression stockings, pneumatic compression boots, UFH, LMWH or warfarin. Compared to the control group, more patients in the intervention group received mechanical prophylaxis (10.0% versus 1.5%, *P* < 0.001) or pharmacologic prophylaxis (23.6% versus 13.0%, *P* < 0.001). The primary end point occurred in 61 patients (4.9%) in the intervention group, as compared with 103 (8.2%) in the control group.[41] In this study, the computer alert reduced the risk of DVT or PE at 90 days by 41% (hazard ratio: 0.59; 95% confidence interval: 0.43–0.81; *P* = 0.001). Though such computerized program might not be available for routine use, a simplified risk assessment tool linked to a specific prophylaxis method can be incorporated into a standard pre-printed admission order, can serve as a reminder for physicians to address this issue at the first encounter.

## Conclusions

The risk of VTE in acutely ill medical patients is well established. Multiple clinical studies have shown the benefit of VTE prophylaxis in this group of patients. Despite consensus recommendations, less than 40% of medically ill patients are actually receiving such prophylactic measures. Strategies to improve prophylactic rate in medical patients are highly needed. Establishment of VTE prophylaxis multidisciplinary team addressing this issue supported by hospital administration is highly needed.
